# Unsatisfactory Neurological Outcome in an Intramedullary Thoracic Intermediate-Grade Melanocytoma—Systematic Review and Illustrative Case

**DOI:** 10.3390/cancers16101867

**Published:** 2024-05-14

**Authors:** Marco Battistelli, Fulvio Grilli, Alessandro Rapisarda, Michele Di Domenico, Nicola Montano, Marco Gessi, Alessandro Olivi, Alessio Albanese, Filippo Maria Polli

**Affiliations:** 1 Department of Neurosurgery, Fondazione Policlinico Universitario “A. Gemelli” IRCCS, 00168 Rome, Italy; marco.battistelli01@icatt.it (M.B.); fulviovincenzo.grilli01@icatt.it (F.G.); michele.didomenico@policlinicogemelli.it (M.D.D.); nicola.montano@policlinicogemelli.it (N.M.); alessandro.olivi@policlinicogemelli.it (A.O.); alessio.albanese@policlinicogemelli.it (A.A.); filippomaria.polli@policlinicogemelli.it (F.M.P.); 2 Neuropathology Unit, Fondazione Policlinico Universitario “A. Gemelli” IRCCS, Università Cattolica S. Cuore, 00168 Rome, Italy; marco.gessi@policlinicogemelli.it

**Keywords:** systematic review, case illustration, intramedullary melanocytoma, spinal tumors, spinal oncology

## Abstract

**Simple Summary:**

Intramedullary spinal cord melanocytomas are exceedingly rare. Here, we present a systematic review of the available literature and a case illustration from our surgical experience: a T1-weighted hyperintense lesion emerges with a focal contrast-enhancing nodule. Despite their benign histology, these tumors often present local aggressiveness with a high tendency towards local invasiveness, which is associated with frequent post-operative neurological deficits, and relapse, which eventually overwhelms adjuvant therapies, such as RT or salvage surgery. We present a case illustration of an intramedullary thoracic melanocytoma in a middle-aged woman. Despite the employment of complete intraoperative neuromonitoring and a frozen section, the adherence of the lesion to the surrounding spinal cord made it impossible to preserve neurological functioning. The current systematic review and case illustration highlight radiographic findings suspected of intramedullary melanocytoma, present the available surgical techniques, and address the importance of preserving neurological functions during the surgical treatment of intramedullary melanocytomas.

**Abstract:**

Background: Intramedullary melanocytomas are exceedingly rare, with only twenty-four cases reported up to now. They present as local invasive tumors despite their benign biological behavior. Attempting a complete safe resection often results in severe post-operative neurological deficits, as in our case presented here. Methods: A systematic review was conducted across the PubMed and Scopus databases including studies published till February 2024. Results: A total of 19 studies were included, encompassing 24 cases. A similar distribution between sexes was noted (M:F 13:11), with ages ranging from 19 to 79 years. The thoracic segment was most affected, and intermediate-grade melanocytoma (19 cases) was the most common histotype. Radiographically, intramedullary melanocytomas usually appear as hyperintense hemorrhagic lesions peripheral to the central canal with focal nodular enhancement. Intraoperatively, they are black–reddish to tan and are tenaciously adherent lesions. In the sampled studies, IONM employment was uncommon, and post-operative new-onset neurological deficits were described in 16 cases. Adjuvant RT was used in four cases and its value is debatable. Recurrence is common (10 cases), and adjuvant therapies (RT or repeated surgery) seem to play a palliative role. Case presentation: A 68-year-old woman presented with a three-year history of worsening spastic paraparesis and loss of independence in daily activities (McCormick grade 4). An MRI revealed an intramedullary tumor from Th5 to Th7, characterized by T1-weighted hyperintensity and signs of recent intralesional hemorrhage. Multimodal neuromonitoring, comprising the D-Wave, guided the resection of a black–tan-colored tumor with hyper-vascularization and strong adherence to the white matter. During final dissection of the lesion to obtain gross total resection (GTR), a steep decline in MEPs and D-Wave signals was recorded. Post-operatively, the patient had severe hypoesthesia with Th9 level and segmental motor deficits, with some improvement during neurorehabilitation. Histopathology revealed an intermediate-grade melanocytoma (CNS WHO 2021 classification). A four-month follow-up documented the absence of relapse. Conclusions: This literature review highlights that intramedullary T1 hyperintense hemorrhagic thoracic lesions in an adult patient should raise the suspicion of intramedullary melanocytoma. They present as locally aggressive tumors, due to local invasiveness, which often lead to post-operative neurological deficits, and frequent relapses, which overwhelm therapeutic strategies leading to palliative care after several years.

## 1. Introduction

Primary tumors of the spinal cord are relatively rare, constituting approximately 2% to 10% of all central nervous system (CNS) tumors, of which 2% to 5% are intramedullary [1,2]. Primary melanocytomas of the CNS are exceptionally rare, with an estimated incidence of approximately one case per million, originating from leptomeningeal scattered melanocytes, typically located at the cranial base, ventral medulla, and cervical spinal cord [3,4]. Because of their embryological origin, they tend to manifest as intradural extramedullary lesions. Brat et al. initially classified CNS melanocytic tumors into low-grade melanocytomas, intermediate-grade melanocytomas, and melanomas based on their histopathological features [5]. With the advent of molecular analyses, missense mutation in codon 209 of the GNAQ gene was defined as a pathognomonic mutation in CNS melanocytic tumors [6]; thus, the recent WHO 2021 classification of CNS tumors took into account histological and molecular features for the classification of CNS melanocytic tumors [7]. Intramedullary localization is exceptionally uncommon, with only twenty-four reported cases in the existing literature. While most ependymomas exhibit a variable surgical cleavage plane, allowing complete resections without significant damage to the spinal cord parenchyma, intramedullary melanocytomas typically present with invasive characteristics which make the dissection troublesome and new-onset neurological deficits at the end of the procedure common [8,9]. Therefore, preoperative radiological suspicion of an intramedullary low- or intermediate-grade melanocytoma prompts the adoption of a tailored surgical approach with the aim of a maximal safe resection. Misdiagnosis may result in inappropriate surgical planning. Here, we present a systematic review of the literature available on intramedullary melanocytomas. The aim is to discuss the radiographic features, macroscopic appearance, intraoperative findings, and biological behavior of these tumors. Finally, we aim at providing insights for the surgical management of intramedullary melanocytoma. An illustrative case treated by our team is also presented, with the aim of offering valuable tips and highlighting possible sources of mistakes.

## 2. Materials and Methods

Literature research was carried out and studies’ eligibility was assessed according to the Preferred Reporting Items for Systematic reviews and Meta-Analyses (PRISMA) guidelines [10]. The systematic review has not been registered. An online literature search was launched on the PubMed/Medline and Scopus databases using the following search string: “(Spinal OR vertebral) AND (intramedullary) AND (melanocytoma OR melanocytic)”; the latest sampled research was published in February 2024. Two authors (F.G. and A.R.) independently conducted abstract screening for eligibility. Any discordance was solved by consensus with a third, senior author (N.M.). No restrictions on date of publication were made. Studies that described intramedullary melanocytoma were included, independently of study design. The exclusion criterion was studies published in languages other than English. A systematic abstract screening of the references (forward search) was performed in order to identify additional records.

## 3. Results

**Figure 1:**
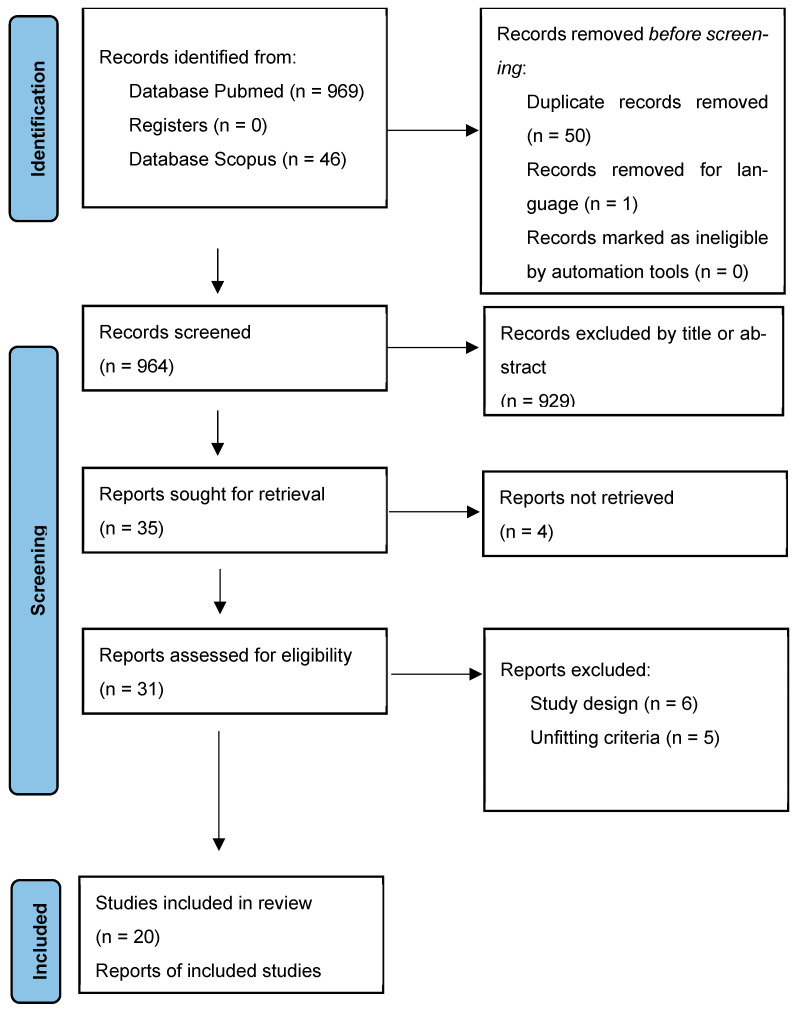
PRISMA flowchart.

The search of the literature yielded a total of 1015 results. Duplicate records were then removed (n = 50). A total of 964 studies were screened, and 929 records were excluded via title and abstract screening; 31 studies were found to be relevant to our research question. Upon full-text review, 20 articles were included in the review, including 25 patients (Figure 1). A summary of results regarding age, sex, extension level, pathology, MRI main characteristics, macroscopic aspect, local aggressiveness, extent of resection, tumor relapse, use of adjuvant RT, use of IONM and its variations, and post-operative neurological examination is available in Table 1.

## 4. Illustrative Case

### 4.1. Clinical History

A 68-year-old woman presented to our outpatient clinic with a three-year history of spastic paraparesis, which had significantly worsened in the year leading up to her admission, rendering her unable to carry out her daily activities (McCormick grade 4). She presented several signs of upper motor neuron dysfunction, including bilateral Babinski reflexes, Achilles clonus, and hyperactive deep tendon reflexes in her lower limbs. Remarkably, she had not a personal or family history of genetic syndromes or previous tumors in other locations.

### 4.2. Imaging Examination

**Figure 2:**
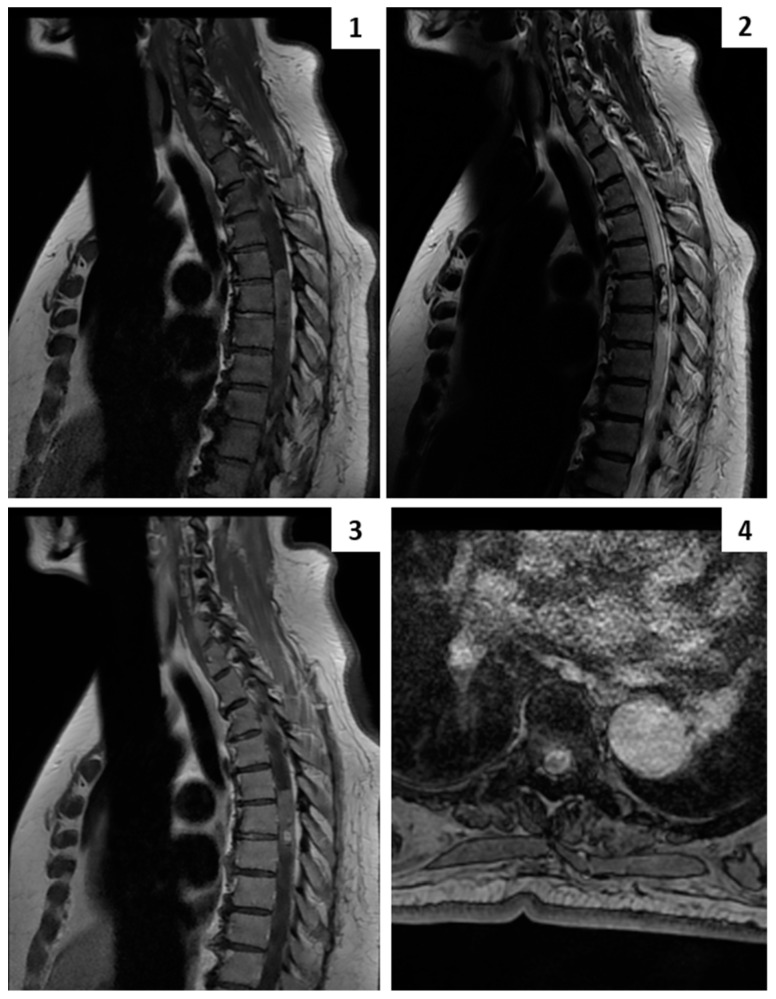
Thoracic preoperative MRI. (**1**) sagittal T1-weighted sequence, (**2**) sagittal T2-weighted sequence, (**3**) sagittal contrast-enhancing T1-weighted sequence, (**4**) axial T1-weighted BRAVO sequence show a thoracic intramedullary tumor extending from Th5 to Th7characterized by hyperintensity in T1-weighted images, an enhancing nodule in CE T1-weighted images, and a heterogeneous aspect in T2-weighted images.

A preoperative total neuraxis MRI revealed an intramedullary tumor spanning from the lower end of Th5 to the upper end of Th7. The lesion exhibited focal hyperintense areas in T2-weighted images, diffuse hyperintensity in T1-weighted images, and focal post-contrast enhancement, notably a 5 mm nodule at the lower end of the tumor. Additionally, significant cord edema was evident, extending several levels both cranially and caudally to the lesion. Furthermore, T1-weighted images indicated recent intralesional hemorrhage (Figure 2).

### 4.3. Management

**Figure 3:**
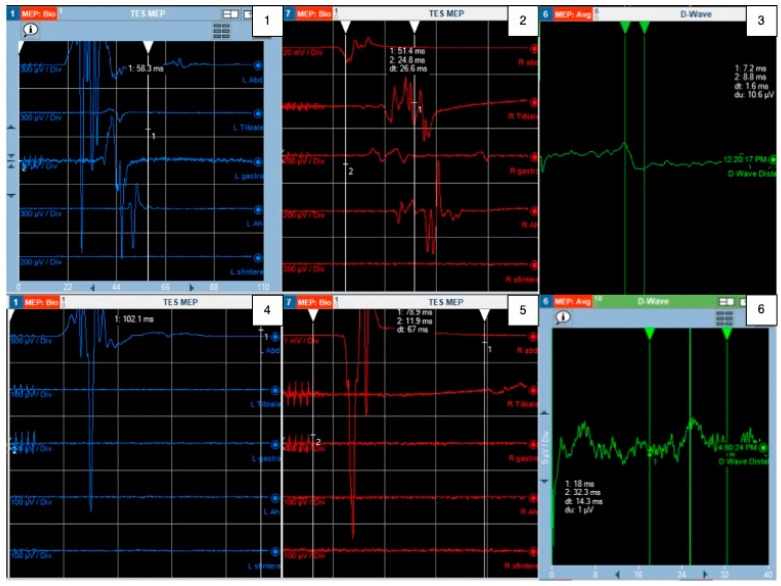
MEPs and D-wave variations throughout surgery. Baseline (**1**) left and (**2**) right inferior limb and sphincter MEPs; (**3**) baseline D-Wave; a morphologically normal D-Wave appears at 10.6 μV stimulation amplitude and with 7.2 ms latency. During tumor dissection from the surrounding spinal cord, sudden disappearance of (**4**) left and (**5**) right inferior limb MEPs and (**6**) D-Wave.

After general anesthesia induction, the patient was placed in the prone position and underwent multimodal neuromonitoring. A midline thoracic skin incision, followed by subperiosteal dissection and a laminectomy spanning Th5-Th6-Th7, was executed, while maintaining continuous neuromonitoring (MEP/SSEP/EMG). Before dura opening, a D-Wave electrode was inserted in the epidural space caudal to the lesion. A careful midline opening of the posterior midline sulcus was performed in order to preserve the gracile fasciculus on both sides. The tumor appeared black–tan-colored and hyper-vascularized and was strongly adherent to the adjacent white matter, making the dissection very difficult. A frozen section biopsy was positive for a potential melanocytic tumor. Given its invasiveness, piecemeal resection was carried out until complete removal was achieved. During the final dissection of the tumor off the white matter, a sudden decline in MEP and D-Wave signals was observed (Figure 3), partially recovered with saline irrigation, corticosteroid administration, and an increase in systemic arterial blood pressure. Despite the best anesthesiological and surgical management, after an initial partial recovery and a stop-and-go surgical strategy, the D-Wave exhibited a further and irreversible drop (Figure 2). Considering the apparent complete resection of the lesion despite the IONM variations, the procedure proceeded with meticulous watertight dural suturing (Supplementary Material—illustrative video). Post-operative dorsal MRI confirmed gross total resection (GTR) of the lesion, while a brain MRI and dermatological examination were unremarkable. The patient exhibited severe hypoesthesia with the Th9 level and significant motor deficits, including thigh abduction/adduction (mMRC 2/5), thigh flexion (mMRC 1/5), leg flexion–extension (mMRC 1/5), and feet flexion–extension (mMRC 1/5). Considered the GTR and the low mitotic index, a radiotherapeutic examination did not indicate a need for adjuvant treatment. An eight-month follow-up total neuraxis revealed the absence of early relapse and confirmed the GTR. During the neurorehabilitatory care, the patient exhibited a slight improvement in segmental motor function, but she was still non-independent in her daily activities (McCormick grade 4). Despite that, at the last follow-up outpatient examination, the patient expressed her general satisfaction because her independence in daily activities had not worsened compared to her preoperative status, and local tumor control had been achieved. She also gave her consent for the present case report.

### 4.4. Histopathology and Immunohistochemistry

Histopathological examination revealed a meningeal neoplasm characterized by cells exhibiting eosinophilic cytoplasm, melanin pigment, rounded nuclei, and focal atypia, along with invasion into the surrounding nervous tissue. Importantly, there was no significant mitotic activity or necrosis detected. Immunohistochemistry displayed positivity for S100, SOX10, Melan A, HMB45, and MITF, while BRAFV600E was negative. Ki67 staining indicated a low proliferation rate of 1–2%. These findings were consistent with the diagnosis of primary meningeal melanocytoma (CNS WHO 2021).

## 5. Discussion

Meningeal melanocytomas are categorized as circumscribed melanocytic lesions of the CNS, further classified into a low grade and intermediate grade based on the mitotic count and/or CNS invasion, as per the WHO 2021 classification of CNS tumors. Low-grade melanocytomas are defined by an absence of mitoses, necrosis, and CNS invasion, while intermediate-grade melanocytomas are defined by a mitotic count of 0.5–1.5 mitoses/mm^2^ and/or evidence of CNS invasion without necrosis, with molecular features including GNAQ, GNA11, PLCB4, or CYSLTR2 mutations [7]. Intramedullary localization is exceedingly rare, with only twenty-four cases reported to date. Analyzing the reported cases reveals a balanced sex distribution (M:F ratio 13:11), with ages at diagnosis ranging from 19 to 79 years. Notably, only three patients were younger than 30 at diagnosis. The melanocytomas are primarily located in the thoracic segment (nineteen out of twenty-four cases), with the remaining five to date localized in the cervical tract (Table 1). Histopathologically, only four cases were described as low grade, while the remainder were intermediate grade.

As for their radiographic features, suspicion of an intramedullary melanocytic lesion can arise from T1-weighted non-enhanced sequences: an hyperintense hemorrhagic lesion peripheral to the central canal should prompt suspicion of a melanocytic tumor in the differential diagnostic process. Additionally, focal nodular enhancement can strengthen the suspicion, while T2-weighted sequences typically show surrounding cord edema or occasional syringomyelia. Only in the case described by Dorwal et al. [4] was contrast enhancement not observed after administration, and, in that case, the authors described a hemorrhagic lesion at the time of MRI acquisition, which could have obscured the contrast enhancement. More common, intramedullary glial-line and mesenchymal vascular tumors exhibit notable radiographic differences from intramedullary melanocytoma. Ependymoma, the most common adult intramedullary thoracic tumor, often presents homogeneous enhancement, a cap sign, and a central location in the spinal cord [28]. Astrocytomas display poorly defined borders and patchy enhancement [28]. Cavernous intramedullary malformations present a pathognomonic appearance in T2-weighted images, with hemosiderin deposition producing a heterogeneous hyperintense core circumscribed by a hypointense rim [29]. Intramedullary hemangioblastomas appear as nodular lesions within a cyst, featuring homogeneous enhancement and distinctive flow voids [30].

Macroscopically, intramedullary melanocytomas are commonly described as black–reddish to tan lesions. Despite their histological benignity, they often exhibit a challenging adherence or invasiveness to surrounding tissue. However, some exceptions are reported. Muthappan et al. [24] described a cervical intramedullary melanocytoma with a good cleavage plane, allowing en bloc tumor removal. In a case reported by Chacko et al. [20], a twenty-two-year-old man with an intramedullary thoracic melanocytoma was noted to have a cleavage plane with respect to the surrounding nervous tissue for most of its perimeter, except at its poles, where it invaded the spinal cord. Despite the presence of a good surgical cleavage plane, both authors reported a considerable decline in motor strength after the operation. However, in the majority of cases, these tumors display strong adherence to the surrounding spinal cord. The neurological post-operative outcome is frequently poor, independent of the presence of a cleavage plane. In light of their rich vascularization, vascular damage during dissection from the nearby CNS may be the source of post-operative neurological deficits.

Given the poor neurological outcomes, the use of cutting-edge technologies to ensure patients’ safety should be considered in the surgical planning. Surprisingly, only seven authors employed complete IONM, with three reporting variations during surgery. Specifically, they reported a worsening of MEPs during the procedure, with disappearance in two cases, which correlated with a worsening of motor functions [16,19]. However, in thirteen cases in which IONM was not employed, a frequent post-operative worsening of neurological function was reported as well. Flores et al. [18] reported an unspecified decrease in MEPs during the operation, leading to the decision to interrupt debulking to preserve neurological functioning. Post-operatively, the patient experienced temporary worsening of left leg strength, which recovered after three weeks, and improved right leg strength. However, the lack of long-term follow-up limited the conclusions that could be drawn on the balance between oncological tumor control and preservation of neurological function in this case. Implementing complete IONM during intramedullary tumor surgery is now considered a routine standard according to NASS 2023 recommendations. In particular, the D-Wave demonstrates the highest positive predictive value in intramedullary lesion surgical resection and is deemed mandatory to consider. IONM enables surgeons to balance functional and oncological outcomes, incorporating stop-and-go surgery to address temporary intraoperative drops in MEPs or the D-Wave [31]. This becomes crucial when surgery is performed for biologically benign tumors with locally aggressive invasive behavior, such as intramedullary melanocytoma. In this context, intraoperative frozen section analysis assumes paramount importance when assessing biological tumor behavior, even though it exhibits the lowest sensitivity (70%) for intramedullary lesions [32].

Regarding oncological disease control, ten authors reported tumor recurrence (Table 1). Of those, six obtained GTR at the end of surgery, while four achieved STR. Nevertheless, four out of six cases of STR experienced a tumor recurrence, while only six out of eighteen cases of GTR had tumor recurrence. Despite the lack of case reports, it appears that GTR protects from tumor relapse. RT was considered in three cases of tumor relapse, of which two were after GTR [12,13,17]. In the case described by Eskandari et al. [12], GTR was achieved of a thoracic intramedullary melanocytoma in a 45-year-old man. Despite that, a three-month FU MRI revealed a local recurrence, which doubled in the following two years. While asymptomatic, fractionated radiation therapy with a total of 5040 cGy was administered. Tumor control was then obtained with stable MRI at the three-year follow-up. Similarly, Wagner et al. described tumor control at eighteen months with fractionated RT administered for a relapse following GTR [13]. However, in the other seven reports, a relapse after GTR was noted during FU MRI, and adjuvant RT was deemed unnecessary [15,17,18,19,21]. In the cases described by Horn et al. [19], despite GTR, recurrence was noted. In two of those cases, adjuvant RT was not undertaken due to the absence of neurological symptoms attributable to the recurrence, and a wait-and-see strategy was preferred. In contrast, a 48-year-old man in whom GTR was achieved experienced tumor recurrence after one year; its growth was observed and he finally underwent a reoperation after eight years. Unfortunately, the patient experienced another relapse after four years with widespread metastasis. At fifteen years after the first surgery, he was referred to palliative care. This case represents the one with the longest follow-up available. Although no conclusions can be drawn in the absence of further studies, it appears that RT and reoperation can achieve only temporary local control of the disease. Irrespective of the extent of the initial resection, intramedullary melanocytoma seems to have a high recurrence rate, ultimately leading to palliative care when treatment strategies are overwhelmed. This, in conjunction with the invasiveness, highlights its locally aggressive behavior despite its benign biology. This may reinforce the notion that less aggressive surgery should be attempted when removing these tumors, with the aim of obtaining the maximal safe resection, as in the case of Flores et al. [18]. This is especially important when considering the advancements in targeted therapies, which may prove effective in treating tumor relapse. On the other hand, preoperative counseling should prepare patients for the eventuality of recurrence and the need for long-term follow-up.

In the case we presented, a 68-year-old woman underwent the resection of an intermediate-grade thoracic melanocytoma. Surgery was performed under multimodal complete IONM and a frozen section was biopsied immediately after tumor exposure, with the aim of detecting neurological deterioration throughout tumor dissection from the surrounding tissues and of balancing the oncological result with function preservation. Despite optimal surgical planning and meticulous surgical techniques, the D-Wave suddenly dropped to 0% with respect to the baseline during tumor dissection from the surrounding white matter. The functional outcome remained unfavorable during neurorehabilitation, highlighting the irreversible nature of CNS damage. The infiltrative nature of the lesion was probably the major factor affecting the negative clinical evolution of the patient.

### Limitations

The main limitation of the present systematic review is the quality of studies included. This limits the possibility of drawing effective conclusions and making assumptions about the reproducibility of the reported data. We propose that a multicenter case series is needed. As for the case illustration, the follow-up to date is limited; we will continue to monitor the patient’s neurological status and radiographic images.

## 6. Conclusions

Intramedullary melanocytomas are exceedingly rare, with our case marking the twenty-fifth reported in the literature. The suboptimal neurological outcome prompted us to engage in a critical reassessment of our strategy, comparing it with previously documented cases to delineate the best practices for managing these tumors. An intramedullary melanocytoma should be considered in the spectrum of differential diagnoses when encountering a T1 hyperintense lesion in the thoracic region with nodular enhancement. Guided by its benign biology, D-Wave variations should inform the resection strategy, aiming for the maximal safe resection, possibly including near-total resection when GTR brings risks of permanent neurological compromise. Despite their benign nature and GTR, these tumors exhibit highly aggressive local behavior due to their invasiveness and high recurrence rates. Adjuvant therapies, whether surgical or radiotherapeutic, can achieve only temporary local control.

## Figures and Tables

**Table 1:** Summary table of available studies on intramedullary melanocytomas. Age, sex, extension level, pathology, MRI main characteristics, macroscopic aspect, local aggressiveness, extent of resection, tumor relapse, use of adjuvant RT, use of IONM and its variations, and post-operative neurological examination are reported.

Author (Year)	Age	Sex	Level	Pathology	Imaging	Macroscopic Aspect	Invasiveness or Adherence (Y/N)	EOR	Relapse (Y/N)	RT (Y/N)	IONM	IONM Variation	Post-op Neurological Worsening (Y/N and What)
Ruggeri, et al. [11] (2014)	74	F	Th7–Th11	Intermediate grade	Hyperintense in T1, hypointense in T2, heterogeneous enhancement	Black and rigid tissue	-	GTR	N	N	MEP, SSEP	-	Y— strength
Dorwal, et al. [4] (2014)	32	F	Th9–Th10	Intermediate grade	Hemorrhagic lesion, not contrast enhancing	Greyish black	Y	GTR	N	N	-	-	N
Eskandari, et al. [12] (2010)	45	M	Th11	Intermediate grade	Hyperintense T1, hypointense T2, contrast enhancing	Reddish mass	-	GTR	Y	Y	MEP, SSEP	-	Y—strength
Hoffmann, et al. [6] (2016)	58	F	Th9–Th10	Intermediate grade GNAQ mutated	Hyperintense T1, contrast enhancing	Grayish red	-	GTR	N	Y	-	-	N
Wagner, et al. [13] (2015)	63	M	C2–C3	Intermediate grade	Hyperintense T1, isointense T2, contrast enhancing	Covered the entire circumference of the spinal cord. Firmly adherent to C2-C3 roots	Y	GTR	Y	Y	MEP, SSEP	-	N
Wang, et al. [9] (2022)	56	M	Th10–Th12	Intermediate grade.	Hyperintense T1, hypointense T2, slight contrast enhancing	Deeply invasive with unclear boundary	Y	STR	N	N	-	-	Y— strength
Karikari, et al. [14] (2009)	20	M	Th12	Low grade.	Hyperintense T2, Contrast enhancing	Fibrotic brown–black mass	-	GTR	N	N	-	-	N
	32	F	Th10	Intermediate grade.	Contrast enhancing	Soft, dark gray lesion	-	GTR	N	N	-	-	Y—strength/bladder/bowel
Liu, et al. [15] (2021)	52	M	Th9–Th10	Intermediate grade.	Hyperintense T1, hypointense T2, heterogeneous enhancement	Well-circumscribed. Without capsule	Y	STR	Y	N	-	-	N
Kim, et al. [16] (2021)	78	M	C3–C5	Low grade.	Hyperintense T1, hypointense T2, slight contrast enhancement	Black pigmented mass with rubbery hard consistency	Y				MEP, SSEP	Upper right arm MEP disappearance	Y—strength/bowel
Covington, et al. [17] (2021)	58	F	foramen magnum-C2	GNAQ +	Contrast enhancing	-	-	GTR	Y	N	-	-	Y—strength/sensitivity/bladder
	70	M	Th9–Th12	Intermediate grade. GNA11 mutated	Contrast enhancing	-	-	STR	Y	Y	-	-	Y— bowel
	66	M	Th12	GNAQ mutated	Contrast enhancing	-	-	GTR	N	N	-	-	Y— sensitivity
Flores, et al. [18] (2021)	69	M	Th8–Th9	Intermediate grade. GNA11 p Q209L mutation.	NO RM (pacemaker)	No clear and dissectible borders	Y	STR	Y	N	MEP, SSEP	Decreased (not specified)	Y— strength
Horn, et al. [19] (2008)	37	F	C1–C3	Low-grade.	Hyperintense T1, isointense T2, contrast enhancing, edema	Cystic component. Black lesion	-	GTR	Y	N	-	-	Y— strenght
	37	F	Th9–D10	Intermediate grade.	Hyperintense T1, hypointense T2, contrast enhancing	Grayish tan	Y	GTR	Y	N	MEP, SSEP	MEP disappearance	Y— strenght
	48	M	Th12	Intermediate grade.	Hyperintense T1, hypointense T2, contrast enhancing	Very dark colored	Y	GTR	Y	N	-	-	Y— strength/ sensitivity
Chacko, et al. [20] (2008)	22	M	Th6–Th11	Low grade.	Hyperintense T1, hypointense T2, contrast enhancing associated with large syrinx	Pigmented tumor. Good cleavage plane except for poles	Y	STR	N	-	-	-	Y— strenght
Perrini, et al. [21] (2010)	79	F	Th10–Th11	Intermediate grade.	Hyperintense T1, hypointense T2, contrast enhancing	Soft and extremely dark	Y	STR	Y	N	-	-	N
Caruso, et al. [22] (2009)	62	M	Th11	Intermediate grade.	Hyperintense T1, hypointense T2, contrast enhancing	Black and rigid	-	GTR	N	N	SSEP	-	Y— Not specified
Abbas, et al. [23] (2023)	35	M	Th10–Th11	Intermediate grade.	Hyperintense T1, hypointense T2, pseudomeningocoele	Compact hardened nidus of black-colored malformed vessels	-	GTR	N	N	-	-	N
Muthappan, et al. [24] (2012)	61	F	C3–C4	Intermediate grade	Hypointense T1, hyperintense T2	Firm, tan-colored, and vascular	N	GTR	N	N	-	-	Y— strenght
Kurokawa, et al. [25] (2013)	40	F	Th10	Intermediate grade	Hyperintense T1, hypointense T2, partially cystic	Black-colored tumor	Y	GTR	N	N	-	-	Y— sensitivity
Turhan, et al. [26] (2004)	19	F	Th8	Intermediate grade	Hyperintense T1, hypointense T2, contrast-enhancing, syringomyelia cavities	Black, rigid, encapsulated	N	GTR	N	N	-	-	-
Ganesan et al. [27] (2017)	34	M	Th11–Th12	Low-grade melanocytoma	Hyperintense T1, hypointense T2, uniform enhancement	Dark black	N	GTR		N	N	-	N

F: female; M: male; Th: thoracic; C: cervical; GTR: gross total resection; STR: sub-total resection; MEP: motor-evoked potential; SSEP: somato-sensory-evoked potential; IONM: intraoperative neuromonitoring; RT: radiation therapy; EOR: extent of resection.

## Supplementary Materials

The following supporting information can be downloaded at https://www.mdpi.com/article/10.3390/cancers16101867/s1, intraoperative video.
